# Money matters: does the reimbursement policy for second-generation antipsychotics influence the number of recorded schizophrenia patients and the burden of stigmatization?

**DOI:** 10.1007/s00127-013-0763-2

**Published:** 2013-09-11

**Authors:** Andrzej Kiejna, Blazej Misiak, Marta Zagdanska, Jaroslaw Drapala, Patryk Piotrowski, Dorota Szczesniak, Sylwia Chladzinska-Kiejna, Magdalena Cialkowska-Kuzminska, Dorota Frydecka

**Affiliations:** 1 Department of Psychiatry, Wroclaw Medical University, 10 Pasteur Street, 50-367 Wrocław, Poland; 2 Institute of Information Science and Engineering, Wroclaw University of Technology, 10 Wyspianski Street, 50-370 Wrocław, Poland

**Keywords:** Schizophrenia, Psychotic disorders, Restricted reimbursement policy, Second-generation antipsychotics, Stigmatization

## Abstract

**Purpose:**

In Poland, non-compliance with the reimbursement policy for second-generation antipsychotics (SGA) manifested in prescribing SGA for patients with psychotic disorders other than schizophrenia may result in serious financial penalties. In this study, we aimed at investigating whether the implementation of the reimbursement policy for SGA contributed to increasing the number of patients with a diagnosis of schizophrenia relatively to the number of patients with a diagnosis of other psychotic disorders in outpatient clinics.

**Methods:**

We analyzed data from *Yearbooks of Mental Health* that were published by the Institute of Psychiatry and Neurology, Warsaw, Poland in the years 1989–2009 registering the number of patients treated for various mental disorders in public facilities in Poland. Temporal trend analysis of the annual number of patients with a diagnosis of psychotic disorders, who were treated at outpatient clinics, was performed.

**Results:**

We found a statistically significant increase in the total number of recorded schizophrenia patients treated at outpatient clinics, as well as in the number of patients treated for the first time at outpatient clinics for schizophrenia. These changes overlap with the implementation of the reimbursement policy for SGA.

**Conclusion:**

Our results suggest that the restricted reimbursement policy for SGA altered the diagnosing process in Poland. It seems that these alterations may have serious social consequences. Given that a diagnosis of schizophrenia is more stigmatizing than a diagnosis of other psychotic disorders, it might be assumed that schizophrenia over-diagnosing, possibly due to reimbursement reasons, add to the enormous burden associated with stigmatization.

## Introduction

Epidemiological data for schizophrenia and other mental disorders are based on reliable medical registers, as well as large population-based and nationwide studies. For instance, the past two decades have provided an immense body of epidemiological studies under the World Mental Health Survey Initiative, which have been based on a methodological consensus [1]. Undoubtedly, the common methodology of these surveys underlies the magnitude of their success and the comparability of results obtained. The last systematic review of the studies on the prevalence of schizophrenia was published by Saha et al. [2]. The authors calculated point, period and lifetime prevalence that equaled 4.6, 3.3 and 4.0 (per 1,000), respectively. Prevalence rates may vary due to research methodological differences, the influence of urbanicity, migrant status or socioeconomic factors [3, 4].

With the ongoing progress in the pharmacotherapy of schizophrenia, in particular with the development of second-generation antipsychotics (SGA), the reimbursement policy for SGA has been gradually implemented in Poland since 1997. Initially, SGA were reimbursed only for patients with ‘treatment-resistant schizophrenia’. However, this term was not clear and became the subject of dispute between psychiatrists and policy makers. In 2004, the Working Group appointed by the Polish Psychiatric Association published a statement explaining the term ‘treatment-resistant schizophrenia’, which was approved by the reimbursement policy makers. A patient with treatment-resistant schizophrenia was defined as anyone who met the ICD-10 criteria for schizophrenia and who did not respond to treatment with at least two first-generation antipsychotics from various chemical groups, which were used in therapeutic doses for at least 4 weeks or such treatment was discontinued due to side effects [5]. The compliance with this definition recorded in patients’ medical documentation allowed to avoid serious financial penalties. Indications for the reimbursement of SGA were extended to all schizophrenia patients and bipolar patients in the years 2010 and 2012, respectively. Currently, all SGA are reimbursed for patients with schizophrenia and selected SGA (aripiprazole, clozapine, quetiapine and olanzapine) are reimbursed for patients with bipolar disorder, while they are fully paid by patients with other psychotic disorders. Although this phenomenon has been widely discussed in the Polish psychiatric community, no action has been taken to change the current reimbursement policy for SGA.

In view of this, one can imagine further consequences of the Polish reimbursement policy. In Poland, patients with a diagnosis of schizophrenia pay less than 10 % of the original price for the treatment with SGA, while patients with other psychotic disorders pay the full price. Therefore, schizophrenia is often diagnosed to provide lower costs of SGA for the patients. This specific Polish reimbursement policy may not only have financial and medical consequences, but also influence the prevalence and incidence rates of various psychotic disorders. Furthermore, given that a diagnosis of schizophrenia is more stigmatizing than other psychotic disorders [6, 7], an increase in the extent of stigmatization might be the core consequence of the reimbursement policy. Stigmatization of schizophrenia patients is still one of the biggest global concerns, although many initiatives have been undertaken to lessen its burden [8, 9]. Therefore, some studies and expert opinions suggest renaming schizophrenia to reduce stigmatization [10–13].

In this study, we aimed at investigating whether the implementation of the reimbursement policy for SGA in Poland influenced the epidemiological figures of psychotic disorders. We analyzed the number of recorded schizophrenia cases along with the number of patients with a diagnosis of other psychotic disorders in the years 1989–2009, based on data sets reported by the Institute of Psychiatry and Neurology, Warsaw, Poland. Our results were partly presented during the 13th Congress of the International Federation of Psychiatric Epidemiology that took place in Taiwan, 30th March–2nd April 2011.

## Methods

### Polish register of mental disorders

We analyzed data from *Yearbooks of Mental Health* that are published by the Institute of Psychiatry and Neurology annually since 1969 [14]. Our analysis was limited to the years 1989–2009. Results from the years 2010–2012 have not yet been published. We included cases with a diagnosis of schizophrenia (F20) and other psychotic disorders (F21–29) according to International Statistical Classification of Diseases and Related Health Problems 10th edition (ICD-10) that were treated at outpatient clinics, including patients treated for the first time (number of patients per 100,000 citizens) in Poland.

The Institute of Psychiatry and Neurology creates the biggest register of patients treated for mental disorders in Poland, based only on data from public facilities including inpatient psychiatric care units, day hospitals and outpatients clinics. In Poland, all public psychiatric facilities are legally obliged to submit data on the number of treated patients with mental disorders to the register created by the Institute of Psychiatry and Neurology. Therefore, data collected in the register are representative with regard to the public setting. This register allows analyzing data with regard to some demographic variables of patients (gender, age, size of the city of residence). Moreover, it provides data on the number of patients treated for the first time for mental disorders. There are also data about the number of mental health facilities as well as the number of health care professionals employed. Data on the number of inpatients with psychotic disorders before the year 1997 were incomplete and unreliable. Therefore, we limited our study to outpatient clinics.

### Statistical analysis

For our statistical analysis, we used MATLAB Statistical Processing Toolbox version r2013a. To analyze whether there were statistically significant changes in the temporal trends of the number of patients with a diagnosis of schizophrenia and patients with a diagnosis of other psychotic disorders treated in the years 1989–2009, we used the following two statistical procedures: one dedicated to detect abrupt changes, and the second one for the detection of continuous changes in the trends. All analyses were performed separately for patients with a history of psychiatric treatment and for those treated for the first time.

**Fig. 1:**
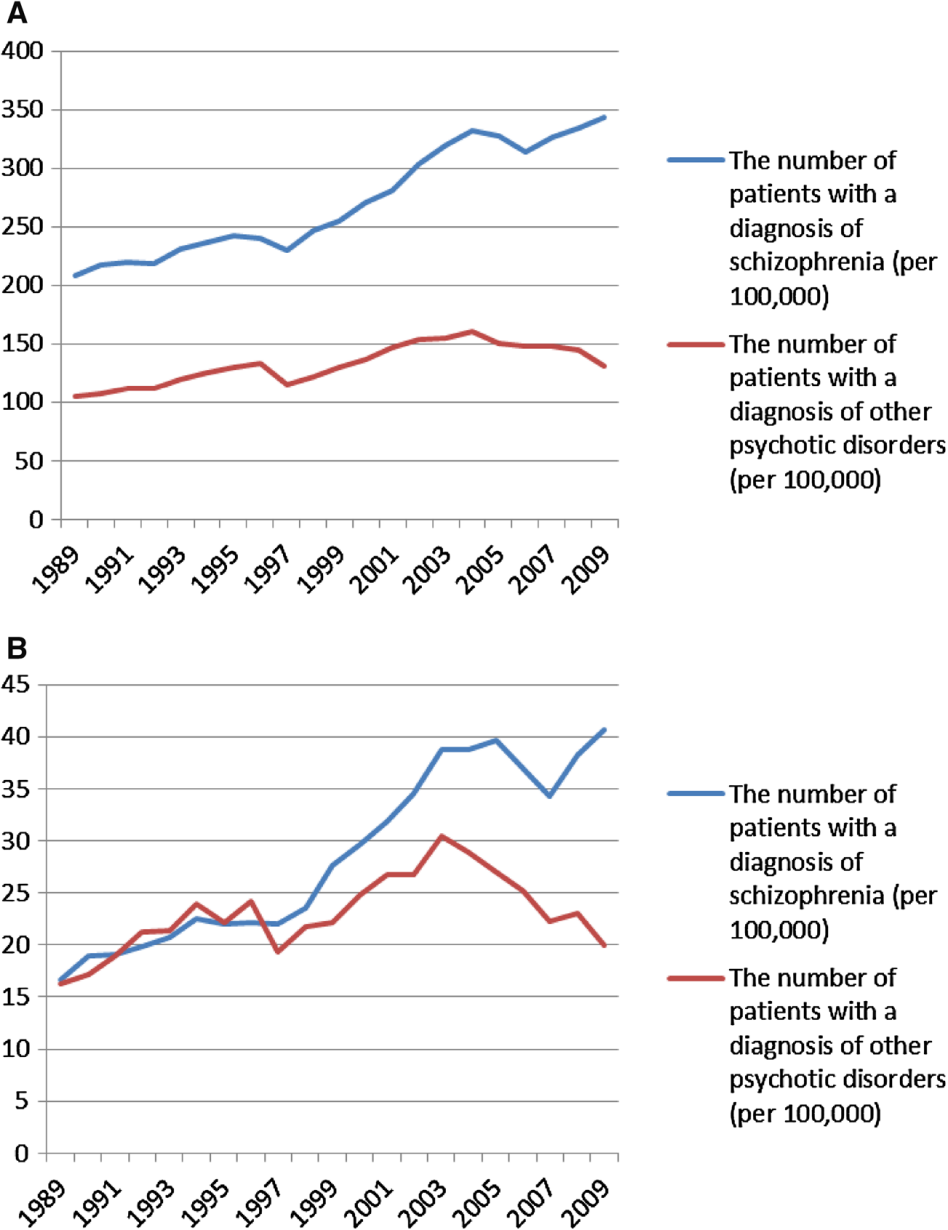
Total number of patients (per 100,000) treated for psychotic disorders (**a**) and the number of patients (per 100,000) treated for the first time for psychotic disorders (**b**)

**Fig. 2:**
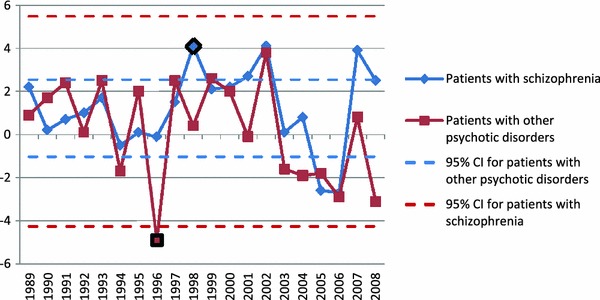
Series of increments in the number of patients with a diagnosis of schizophrenia and other psychotic disorders treated for the first time at outpatient clinics (*95* *% CI* 95 % confidence interval)

**Fig. 3:**
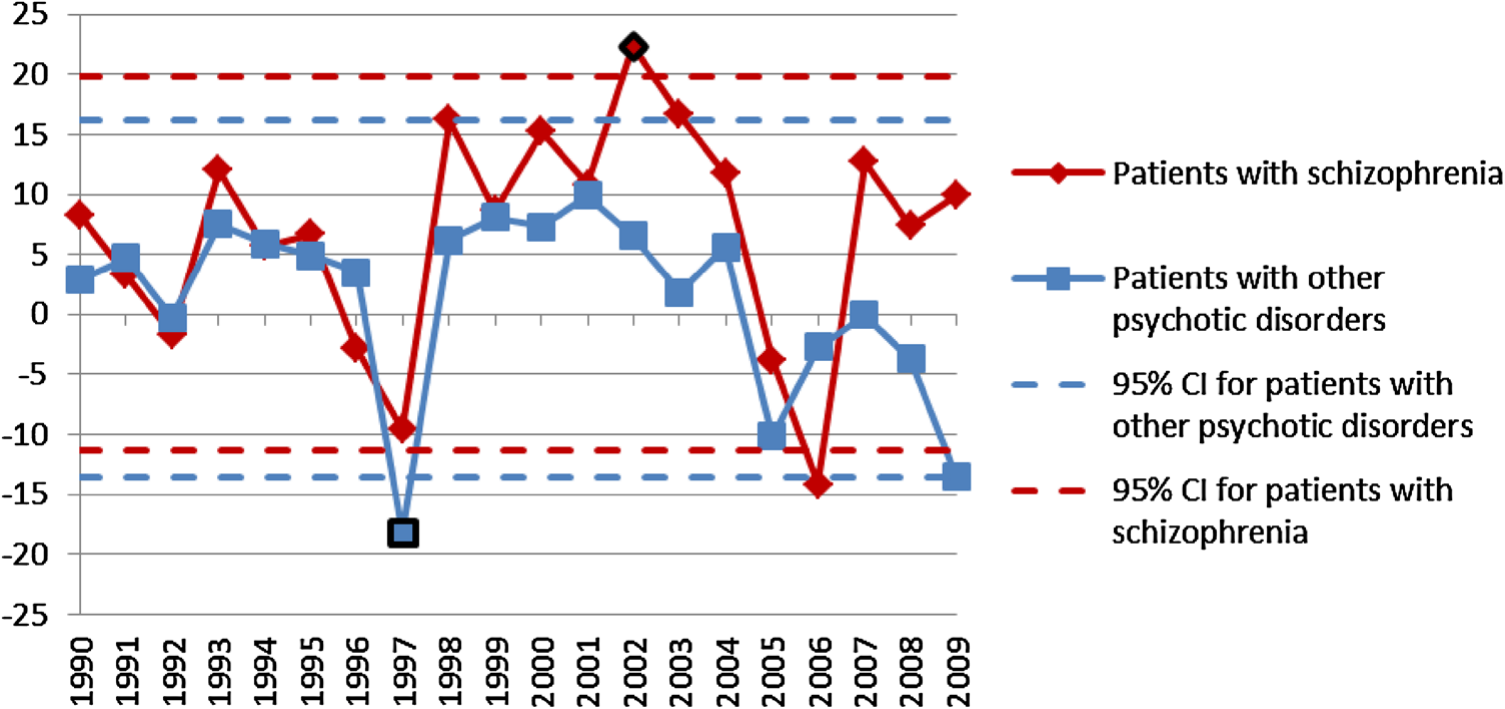
Series of increments in the total number of patients with a diagnosis of schizophrenia and other psychotic disorders treated at outpatient clinics (*95* *% CI* 95 % confidence interval)

Both statistical procedures were based on the series of numbers of patients receiving treatment in a given year at outpatient clinics (total number of patients per 100,000 citizens) for a diagnosis of schizophrenia [$\left( {x_{n}^{\text{scz}} } \right)_{n = 1989}^{2009}$] and other psychotic disorders [$\left( {x_{n}^{\text{others}} } \right)_{n = 1989}^{2009}$] [temporal patterns of $\left( {x_{n}^{\text{scz}} } \right)_{n = 1989}^{2009}$ and $\left( {x_{n}^{\text{others}} } \right)_{n = 1989}^{2009}$ are plotted in Fig. 1a, b for all the patients and patients treated for the first time, respectively]. Based on these data, the series of increments of the number of patients between every two consecutive years were calculated separately for patients with a diagnosis of schizophrenia [$\left( {d_{n}^{\text{scz}} } \right)_{n = 1990}^{2009}$, where $d_{n}^{\text{scz}} = x_{n}^{\text{scz}} - x_{n - 1}^{\text{scz}}$] and with a diagnosis of other psychotic disorders [$\left( {d_{n}^{\text{others}} } \right)_{n = 1990}^{2009} ,$ where $d_{n}^{\text{others}} = x_{n}^{\text{others - }} - x_{n - 1}^{\text{others}}$] [temporal patterns $\left( {d_{n}^{\text{scz}} } \right)_{n = 1990}^{2009}$ and $\left( {d_{n}^{\text{others}} } \right)_{n = 1990}^{2009}$ are plotted in Figs. 2 and 3 for all the patients and the patients treated for the first time, respectively].

To find the time point of the change in the temporal trend of the number of patients, we split the series of data into two parts (“left” for the trend before a given time point and “right” for the trend after a given time point) choosing as a splitting time point consecutively all dates between 1993 and 2007 and looking for the time point (“critical year”) of significant changes between these two parts of the series of data (i.e., “left” and “right”).

In the first statistical procedure looking for the abrupt changes in the trend, the first part of the series of data (“left”) was used to recover the probability density function (PDF) of the sequences of increments for patients with a diagnosis of schizophrenia $\left( {d^{\text{scz}} } \right)$ and other psychotic disorders ($d^{\text{others}}$). Sequences were successfully tested for normality using the Lilliefor’s test (*p* value >0.05), and likelihood estimates of means and variances were calculated. Figures 2 and 3 present 95 % confidence interval (95 % CI) for all the patients and patients treated for the first time, respectively. Finally, one-tailed *Z* test was applied to determine the likelihood of each observation to find the time point (“critical year”) at which a significant abrupt change in the temporal trend in the number of patients can be observed.

**Fig. 4:**
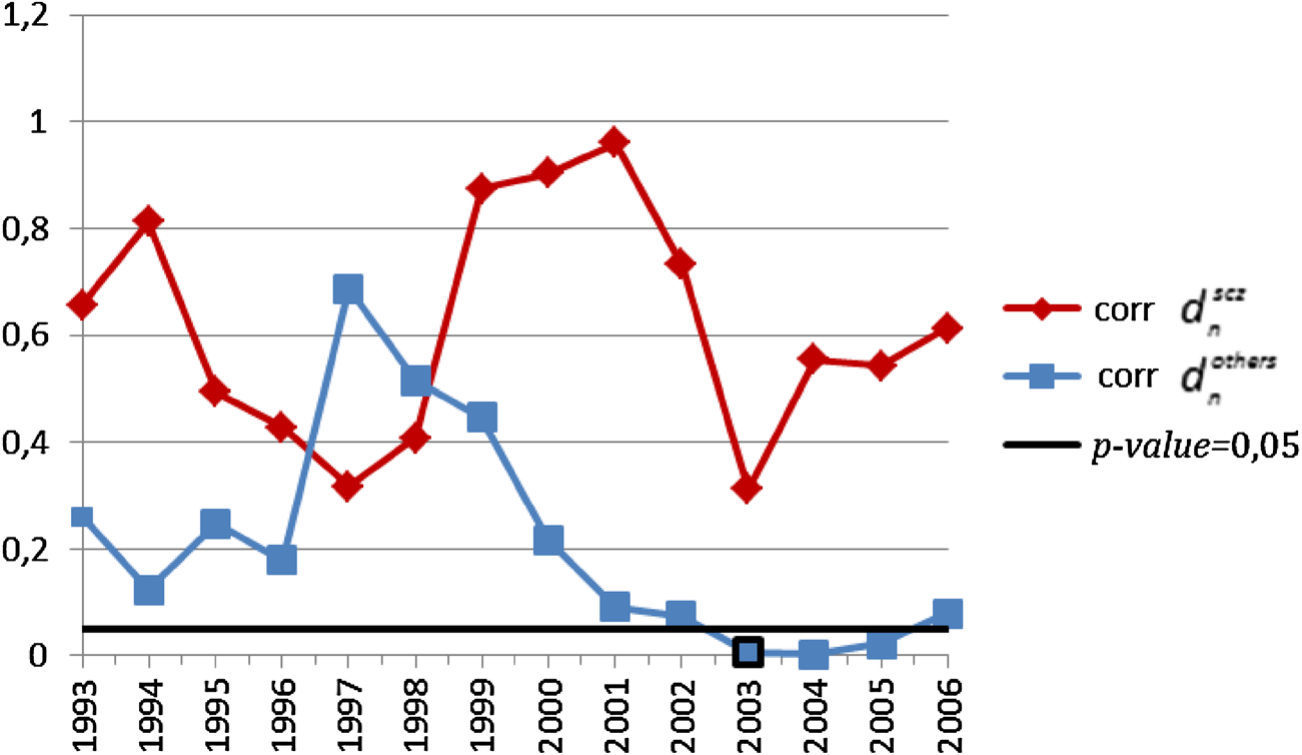
The plot of *p* values of covariance analysis for the number of patients treated for the first time in outpatient clinics for psychotic disorders

**Fig. 5:**
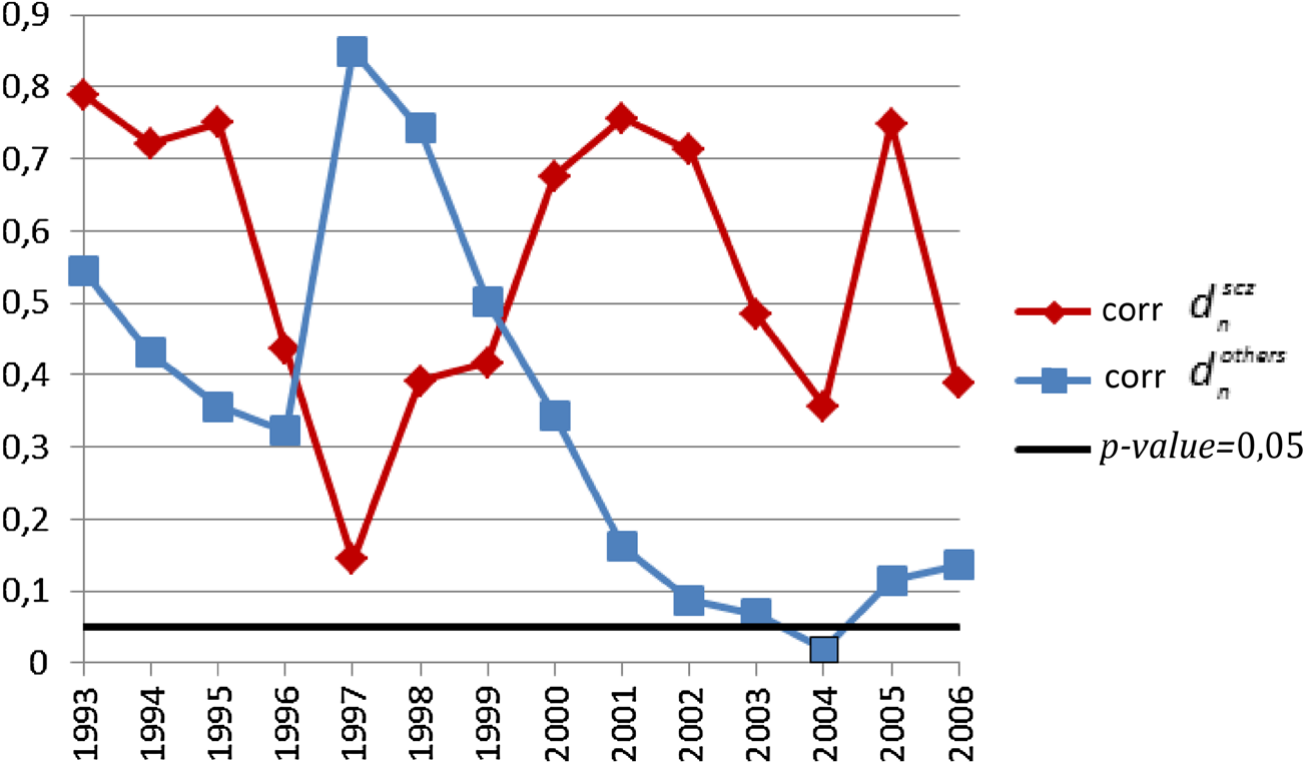
The plot of *p* values of covariance analysis. The covariance analysis for the total number of patients treated for psychotic disorders

In the second statistical procedure looking for continuous changes in the temporal trend, the autocorrelation function [15] was applied (correlation between $d_{n}^{\text{scz}}$ and $d_{n - 1}^{\text{scz}}$ for patients with a diagnosis of schizophrenia and $d_{n}^{\text{others}}$ with $d_{n - 1}^{\text{others}}$ for patients with a diagnosis of other psychotic disorders). The covariance analysis was used to compare trends of the first part of the series of data (“left”) with the second part of the series of data (“right”) to find the time point (“critical year”) at which significant continuous change of temporal trend in the number of patients can be observed (temporal patterns of *p* values of covariance analysis are plotted in Figs. 4 and 5 for the first time-treated patients and the whole group, respectively).

**Fig. 6:**
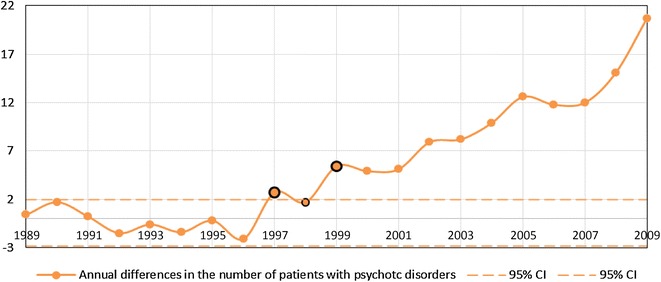
The plots of the sequences of annual differences in the number of patients treated for the first time for psychotic disorders at outpatient clinics (*95* *% CI* 95 % confidence interval)

**Fig. 7:**
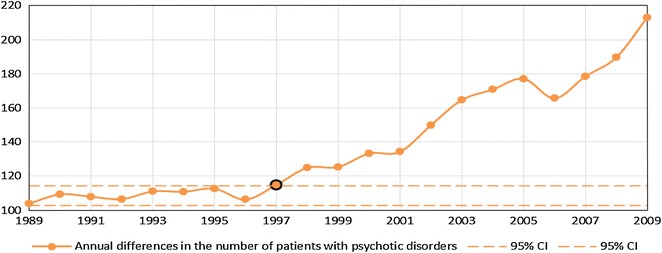
The plots of the sequences of annual differences in the total number of patients treated for psychotic disorders at outpatient clinics (*95* *% CI* 95 % confidence interval)

Additionally, to analyze statistically significant changes in the temporal trend of differences between the number of patients with a diagnosis of schizophrenia and a diagnosis of other psychotic disorders, the annual sequence of differences was calculated ($h_{n} = x_{n}^{\text{scz}} - x_{n}^{\text{others}}$). The first part of the series of these data (“left”) was used to recover the PDF of these sequences of differences [temporal patterns $(h)_{n = 1989}^{2009}$ are plotted in Figs. 6 and 7 for the first time-treated patients and the whole group, respectively]. Sequences were successfully tested for normality using the Lilliefor's test (*p* > 0.05), and likelihood estimates of means and variances were calculated. Finally, one-tailed *Z* test was applied to determine the likelihood of each observation, to find the time point (“critical year”) at which the significant change of the temporal trend of differences in the number of patients with a diagnosis of schizophrenia and other psychotic disorders can be observed.

## Results

Significant differences in the rates of temporal changes in the trend of the annual number of patients with a diagnosis of schizophrenia $\left( {d^{\text{scz}} } \right)$ and patients with a diagnosis of other psychotic disorders ($d^{\text{others}}$) treated at outpatient units were observed. Among patients treated for the first time, in the group of patients with schizophrenia, the significant abrupt change in the trend occurred in the year 1999 (*p* value 0.00012), whereas in the group of patients with other psychotic disorders, in the year 1997 (*p* value 0.013). Among the whole group of patients, in the group of patients with a diagnosis of schizophrenia, significant abrupt change in the trend occurred in the year 2001 (*p* value 0.0051), whereas in the group of patients with a diagnosis of other psychotic disorders, in the year 1997 (*p* value 0.012). Notably, the direction of change was different for these groups: the number of patients with a diagnosis of schizophrenia increased and that of patients with a diagnosis of other psychotic disorders decreased. After these significant time points (“critical years”) of change, the temporal trends for both groups of patients were again stable, however, at a higher level for patients with a diagnosis of schizophrenia and at lower level for patients with a diagnosis of other psychotic disorders (*p* value >0.6). The graphical representations of the series of increments in the number of patients with a diagnosis of schizophrenia and other psychotic disorders in each year together with the time points of abrupt temporal change (“critical years”) are presented in Figs. 2 and 3 (for the patients treated for the first time and the whole group, respectively).

The analysis of continuous changes of temporal trends in the annual number of patients with a diagnosis of schizophrenia and psychotic disorders revealed that among the patients treated for the first time, a statistically significant change in the trend was observed only among patients with a diagnosis of other psychotic disorders and was initiated in the year 2003 (*p* value 0.006). The plot of *p* values of covariance analysis is shown in Fig. 4. Among the whole group of patients already treated in psychiatric facilities, a statistically significant change in the trend was also observed only among patients with a diagnosis of other psychotic disorders—it started in the year 2004 (*p* value 0.019). The plot of *p* values of covariance analysis is shown in Fig. 5.

Assessment of differences between the annual number of patients with a diagnosis of schizophrenia ($x^{\text{scz}}$) and patients with a diagnosis of other psychotic disorders $\left( {x^{\text{others}} } \right)$ revealed a significantly larger difference in the year 1997 (*p* value 0.0054 and *p* value 0.0159 for patients treated for the first time and for those treated already in psychiatric facilities, respectively). The plots of the sequences of annual differences in the number of patients are shown in Figs. 6 and 7. Interestingly, among the patients with a diagnosis of schizophrenia treated for the first time, the year 1998 can also be considered as a significant changing point (*p* value 0.041); however, this result lies slightly below the 95 % CI line, because we used the unbiased estimator of variance to produce 95 % CI interval that makes its range wider.

## Discussion

Our results indicate a significant change in the trend in the number of diagnosed psychotic disorders at outpatient clinics that was initiated in 1997, overlapping with the implementation of the restricted reimbursement policy for SGA. Notably, there was an inverse change in the trend in diagnosing psychotic disorders. It seems that decrease in the number of patients with a diagnosis of other psychotic disorders (ICD-10 categories F21–29) preceded the increase in the number of patients with a diagnosis of schizophrenia that appeared already in 1997. A significant change in the trend in diagnosing schizophrenia occurred in 1999. We hypothesize that these changes are the consequence of the restricted reimbursement policy for SGA that was implemented in 1997. Non-compliance with the reimbursement policy may result in severe financial penalties directed at clinicians. The second change in diagnostic trends was observed in the years 2002–2004, which may have been due to the inspections of compliance with reimbursement restrictions that were particularly common at outpatient clinics and intensified in the years 2002–2004 coinciding with the change in diagnosing psychotic disorders.

Besides the falsification of epidemiological measures, the misdiagnosis of schizophrenia may have severe consequences for patients. It seems that increased burden of stigmatization is the direct and core consequence of the restricted reimbursement policy for SGA, which may trigger the majority of the negative social sequelae. Stigmatization of people with severe mental illness results in lower availability of health care services, poorer quality of management of physical health problems, difficulties in employment and housing and social isolation [16]. Although a great progress in fighting against stigma in psychiatric community was made in the past decade, a diagnosis of schizophrenia still constitutes an enormous burden for patients. In Poland, several local and national anti-stigma initiatives have been conducted in recent years. However, there are no reliable results of these initiatives [17]. Moreover, there is scarcity of studies on stigmatization in psychiatry from Poland. According to the survey that was carried out by Public Opinion Research Center, which is a public opinion agency, 61 % of respondents have negative stereotypes about people with mental disorders [17, 18]. The patient’s perspective reflects the attitudes presented by public opinion. In the study by Cechnicki et al. [19], almost 60 % of patients with schizophrenia anticipated stigmatization, while 87 % experienced stigmatization in social relationships. Therefore, it is not surprising that more than 80 % of schizophrenia patients avoid disclosing their diagnosis outside the closest family members because of the fear of rejection [20]. In view of these findings, a diagnosis of schizophrenia is in itself stigmatizing for the patients. Stereotypes that circumscribe schizophrenia and its consequences serve as the source of self-stigma, which refers to the internalization of mental illness resulting in decreased self-esteem and self-efficacy [21]. On the other hand, it should be kept in mind that the misdiagnosis of schizophrenia may obscure social perception of schizophrenia enhancing negative attitudes toward patients. In view of the increased burden of stigmatization and its consequences, it might be concluded that the restricted reimbursement policy for SGA generates more costs than savings and there is an urgent necessity to extend the policy to other psychotic disorders.

One may criticize the reliability of the Polish register of mental disorders. Medical registers have a long tradition in Nordic countries, and their reliability makes them a valuable and model source of epidemiological data sets. Nordic registers have provided data on, e.g., high mortality in schizophrenia due to natural causes, the role of urbanicity in the etiology of schizophrenia or the role of obstetric complications in the development of schizophrenia [22]. Polish register of mental disorders is characterized by the lack of systemic solutions for data flow from psychiatric facilities. For instance, private psychiatric facilities are not obliged to report established diagnoses. Hence, we may assume that the virtual increase in the number of schizophrenia cases is even higher than that reported in our study. However, the private psychiatric setup constitutes the minority of the Polish mental health care system. Additionally, patients with psychotic disorders are treated mainly at public mental health services. Limitations of the register do not permit providing incidence rates, which are strongly dependent on how reliably the onset is determined. The onset of schizophrenia is particularly difficult to determine. Neither the first hospitalization nor the first appointment at the outpatient clinic is the most reliable definition of the onset. Hence, the first contact with any psychiatric or general health service constitutes a better indicator of the onset [23]. Taking into account these considerations, data from the register conducted by the National Institute of Psychiatry and Neurology do not provide the incidence rates for schizophrenia and other psychotic disorders. Cases which are reported as first hospitalizations or first contacts with outpatient clinics are not synonymous with first-episode cases. Indeed, patients who are hospitalized for the first time could first have had contact with a psychiatrist at public or private outpatient clinics. Conversely, patients who have visited outpatient clinics may have been previously hospitalized for the first time. In this regard, it might be beneficial to utilize the person identifier not only for insurance or health care purposes, but also for register purposes. Although the register is characterized by several limitations, the point prevalence rates were as follows: between 0.21 % in 1989 and 0.34 % in 2009 for schizophrenia, and between 0.10 % in 1989 and 0.16 % in 2004 (Fig. 1a). These prevalence rates are similar to those obtained by Saha et al. [2]. However, some underestimation arising from exclusion of inpatients should be taken into account.

Notably, several factors might also underlie the phenomenon described in our study. These include changes in the availability or accessibility of services, shifting to outpatient care, positive changes in diagnostic practice (e.g., better or earlier identification of cases), a real increase in incidence, improved survival, a change in the proportion of people accessing public versus private health care and a change in the completeness of the records. Availability as well as accessibility of outpatient clinics decreased in the years 1995–2000 (the number of outpatient clinics was 683 in 1995 and 591 in 2000), but increased significantly in the years 2000–2005 (there were 1,187 outpatient clinics in 2005) [24]. It should be noted that this increase in the number of outpatient clinics followed an increase in the number of recorded schizophrenia patients, which started in 1997. Therefore, changes in the availability or accessibility of psychiatric services could only enhance schizophrenia diagnosing and most likely did not initiate this phenomenon. Similarly, existing data do not support the phenomenon of shifting psychiatry to outpatient care in Poland. Both the number of hospitalizations and the number of patients treated at outpatient clinics increased significantly in the years 1995–2007—from 367 to 542 per 100,000 and from 1885 to 3,571 per 100,000, respectively [24]. In turn, diagnostic practice still leaves much to be desired and the National Programme of Mental Health for the years 2011–2015 ultimately emphasizes the necessity of early identification initiatives. We cannot exclude that results obtained might also reflect a real increase in schizophrenia incidence. However, previous meta-analyses and systematic reviews suggest that incidence rates remain stable or may decrease over time [25, 26]. Furthermore, in a recent study by Sutterland et al. [27], no significant time trend of incidence rates of schizophrenia spectrum disorders was found in the Netherlands in the years 1996–2006, which is the time period overlapping with that analyzed in our study. Our results might also be the consequence of changes in survival of schizophrenia patients. To the best of our knowledge, studies on mortality in schizophrenia in Poland have not been performed so far. Another phenomenon that could underlie an increase in the number of schizophrenia patients is a change in the proportion of people accessing public versus private health care. Notably, private outpatient clinics are focused mainly on management of non-psychotic mental disorders. Moreover, first-episode schizophrenia patients are usually treated within public inpatient units. Finally, data flow in the Polish register of mental disorders did not change over time, and thus did not affect the number of recorded schizophrenia patients. Our results are even more interesting in the light of the well-known fact that psychiatrists themselves are reluctant to diagnose schizophrenia because of the stigma burden.

Our study has some limitations that should be addressed. Firstly, we did not assess variables connected to stigmatization. Furthermore, we had not performed the objective analysis of clinicians’ decisions in first-episode patients. We also did not have access to data on the number of SGA prescriptions and costs of reimbursement in the studied period of time. Finally, we did not perform the field study that would provide the proportion of patients who are justly diagnosed with schizophrenia. However, to the best of our knowledge, it is the first study on social and epidemiological consequences of the restricted reimbursement policy for SGA, but our results should be considered as preliminary. It would be also beneficial to perform the study based on the Delphi consensus methodology to gain knowledge on clinicians’ decisions in diagnosing psychotic disorders. Reports from other countries, which implemented similar reimbursement policies, are also required to recognize the extent of the phenomenon described in this article. In conclusion, it should be highlighted that the burden of indirect costs associated with the implementation of restricted reimbursement policies for various pharmacological treatment strategies may exceed the benefits of direct cost reduction. Therefore, restricted reimbursement policies in psychiatry should be implemented with caution and with a wide insight into possible social and economic consequences.
